# Therapeutic Angiogenesis Using Autologous CD34-Positive Cells for Vascular Diseases

**DOI:** 10.3400/avd.ra.22-00086

**Published:** 2022-12-25

**Authors:** Yasuyuki Fujita, Atsuhiko Kawamoto

**Affiliations:** 1 Translational Research Center for Medical Innovation, Foundation for Biomedical Research and Innovation at Kobe, Kobe, Hyogo, Japan

**Keywords:** CD34+ cell therapy, peripheral arterial disease, cardiovascular disease, cerebrovascular disease

## Abstract

CD34 is a cell surface marker, which is expressed in various somatic stem/progenitor cells such as bone marrow (BM)-derived hematopoietic stem cells and endothelial progenitor cells (EPCs), skeletal muscle satellite cells, epithelial hair follicle stem cells, and adipose tissue mesenchymal stem cells. CD34+ cells in BM and peripheral blood are known as a rich source of EPCs. Thus, vascular regeneration therapy using granulocyte colony stimulating factor (G-CSF) mobilized- or BM CD34+ cells has been carried out in patients with various vascular diseases such as chronic severe lower limb ischemia, acute myocardial infarction, refractory angina, ischemic cardiomyopathy, and dilated cardiomyopathy as well as ischemic stroke. Pilot and randomized clinical trials demonstrated the safety, feasibility, and effectiveness of the CD34+ cell therapy in peripheral arterial, cardiovascular, and cerebrovascular diseases. This review provides an overview of the preclinical and clinical reports of CD34+ cell therapy for vascular regeneration.

## Introduction

CD34 cell surface antigen is a single transmembrane phosphoglycoprotein whose molecular weight is approximately 115 kDa. CD34 was first identified in 1984 on hematopoietic stem and progenitor cells (HSPCs).^1)^ Although the CD34 antigen is structurally well investigated and useful in identifying HSPCs, the actual functions of the CD34 antigen have remained relatively elusive. Accumulated studies revealed that CD34 is expressed in various somatic stem/progenitor cells such as endothelial progenitor cells (EPCs), skeletal muscle satellite cells, corneal keratocytes, interstitial dendritic cells, epithelial progenitor cells, and adipose tissue mesenchymal stem cells as well as bone marrow (BM)-derived HSPCs.^2)^ In other words, CD34 is considered to be a marker of various stem cells in vivo. Cells expressing CD34 are referred to as CD34-positive (CD34+) cells.

**Figure 1:**
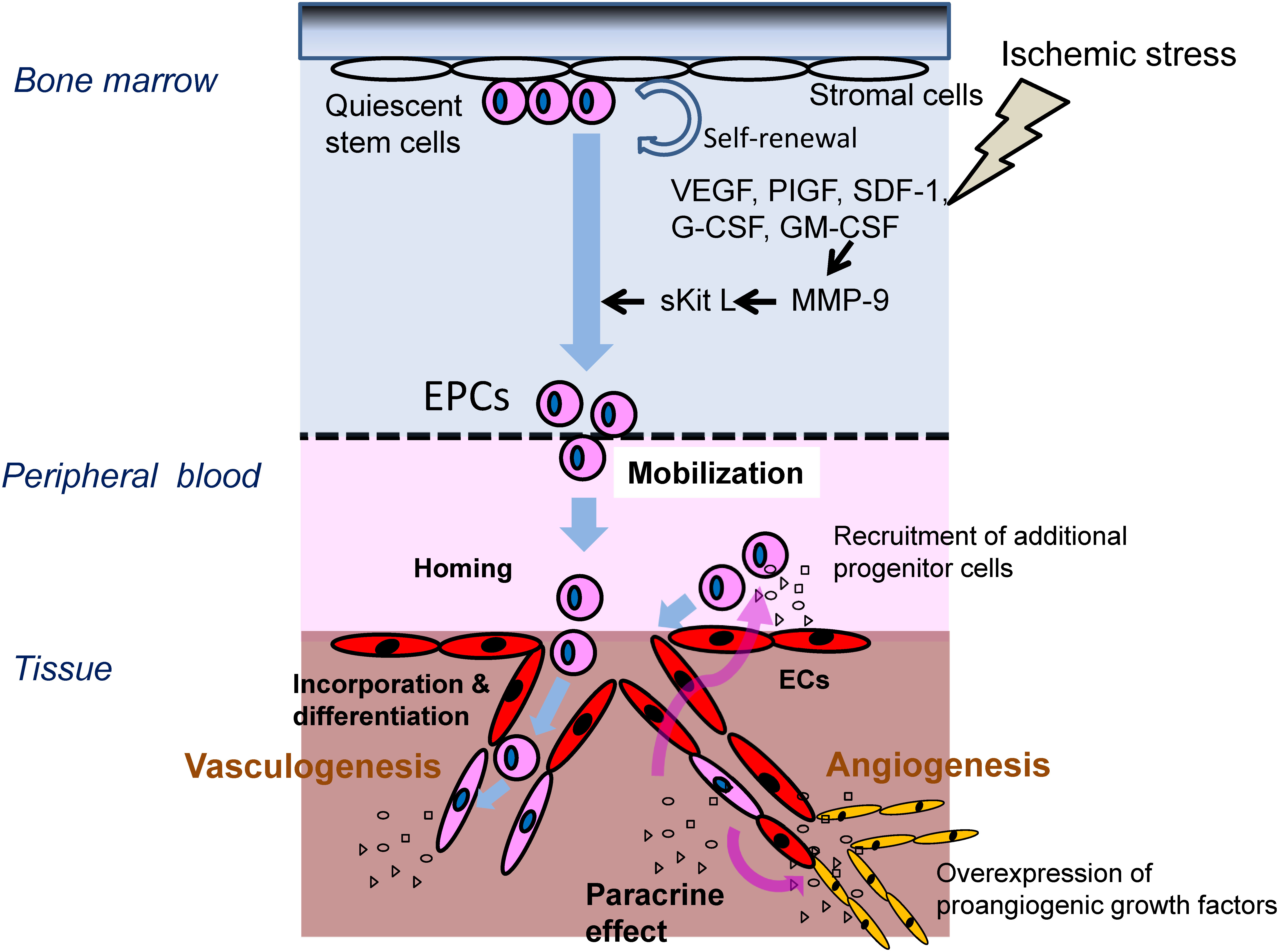
Fig. 1 Kinetics of EPCs.

EC: endothelial cell; EPC: endothelial progenitor cell; G-CSF: granulocyte colony-stimulating factor; GM-CSF: granulocyte macrophage–colony-stimulating factor; MMP-9: matrix metalloproteinase-9; PlGF: placental growth factor; SDF-1: stromal cell-derived factor; sKit L: soluble kit ligand; VEGF: vascular endothelial growth factor

In 1997, EPCs were first identified in adult human peripheral blood (PB) as CD34+ mononuclear cells (MNCs).^3)^ They are phenotypically characterized by the expression of antigens associated with HSPCs including CD133, CD34, c-kit, vascular endothelial growth factor receptor-2, CD144 (vascular endothelial–cadherin), and stem cell antigen-1. The discovery of circulating EPCs changed the traditional paradigm that “vasculogenesis” occurs exclusively in the developing embryo. EPC concentration in the PB is low under normal conditions; however, EPCs residing in the BM are mobilized into PB in response to physiological and pathological stimuli, such as myocardial and peripheral ischemia. Mobilized EPCs recruit to the foci of neovascularization where they form structural components of the growing vasculature. Conversely, Gehling et al.^4)^ reported that cells in PB expressing AC133 (CD133), an undifferentiated hematopoietic stem cell marker, can differentiate into vascular endothelial sequences. As a vascular regeneration mechanism by EPC, besides vascular endothelial development by EPC itself, EPCs were found to produce various cytokines and vascular growth factors involved in angiogenesis, such as vascular endothelial growth factor, basic fibroblast growth factor, angiopoietin-1, hepatocyte growth factor, insulin-like growth factor-1, stromal cell-derived factor-1, and endothelial nitric oxide synthase, promoting the proliferation of the existing vascular endothelium and cellular migration (paracrine effect).^5–7)^ Furthermore, EPCs have been shown to secrete not only angiogenesis-related proteins but also ribonucleic acids and exosomes containing microRNA, which contribute to the paracrine effect via gene control mechanisms (**Fig. 1**).^8,9)^

**Figure 2:**
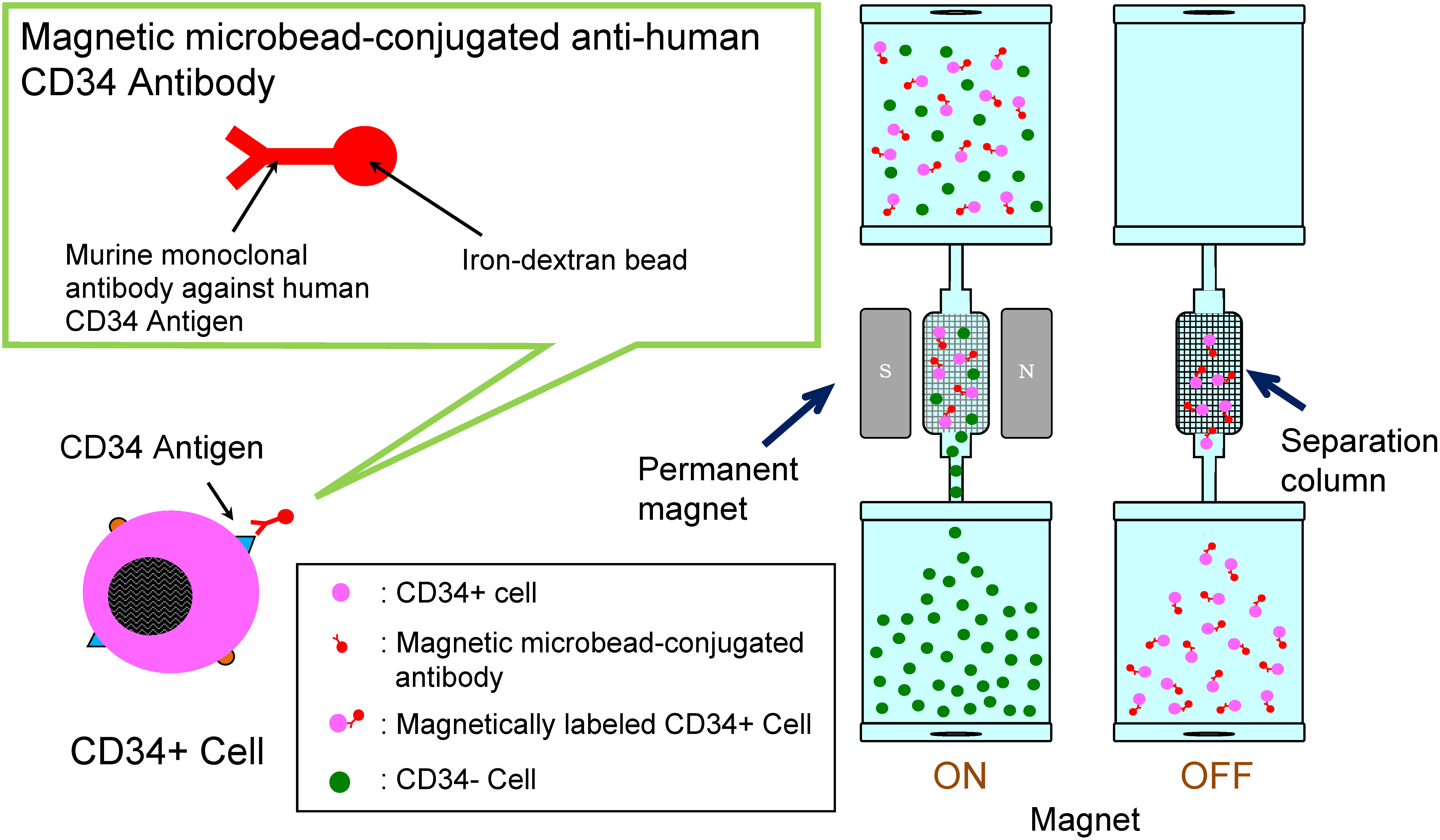
Fig. 2 Fundamental principles of immunomagnetic separation of CD34-positive (CD34+) cells.

The superiority of isolated EPCs over unselected BM- or PB-MNCs as a cell source for vascular regeneration therapy has been demonstrated in several preclinical studies. Yoon et al.^10)^ showed that myocardial calcification occurred with high frequency when whole BM cells were transplanted intramyocardially into the rat model of acute myocardial ischemia. Kawamoto et al.^11)^ demonstrated that intramyocardial transplantation of high-dose PB-MNCs into the rat model of acute myocardial ischemia led to intramyocardial hemorrhage with infiltration of many inflammatory cells and a less improvement in neovascularization and cardiac function. By contrast, transplantation of purified CD34+ cells was associated with an absence of such adverse reactions, high levels of neovascularization, and sustained recovery of cardiac function. The in vitro EPC colony-forming assay developed by Masuda et al.^12)^ showed that EPC colonies are formed from CD34+ cells at a high frequency, whereas EPC colonies could not be obtained from CD34− MNCs even when using 100 times more cells, demonstrating a marked difference in vascularization potential between the EPC and non-EPC fractions. These results suggest that the transplantation of purified EPCs is superior to BM- or PB-MNC transplantation in terms of therapeutic effect and safety. Moreover, CD34+ or CD133+ cell therapy is feasible because a clinical-grade device for immunomagnetic cell separation has already been developed and cell culture is not required in the separation process (**Fig. 2**).

In this review, focusing on BM- or PB-CD34+ cells, we introduce the results of major preclinical and clinical trials of vascular regeneration therapy for various cardiovascular and cerebrovascular diseases.

## Peripheral Arterial Diseases

Peripheral arterial disease (PAD), which is a circulation disorder caused by the stenosis or occlusion of arteries in the limbs, is estimated to affect more than 200 million individuals globally, ranging from asymptomatic to severe.^13)^ The most common underlying disease is arteriosclerosis obliterans (ASO) caused mainly by atherosclerosis; other underlying diseases include thromboangiitis obliterans (TAO) (also known as Buerger’s disease), vasculitis, and autoimmune diseases.

Chronic critical limb ischemia (CLI) is defined as the end-stage PAD, in which patients experience pain at rest and have ulcers or gangrene on their lower limbs (Fontaine classification stage III or more severe or Rutherford classification category 4 or more severe) that persists for 2 weeks or longer. The prognosis of CLI is generally very poor, being comparable with that of some advanced malignancies. The mortality rate is 25%, the ratio of survival after major amputation is 30%, and the CLI persistence rate is 20% at 1 year after diagnosis of CLI. Furthermore, CLI patients have a 5 year survival rate of 40%–50%.^14)^ The annual incidence of CLI is 500–1,000 per million in Europe and the United States.^14)^ An estimated 250,000 amputations of lower limbs were performed annually due to CLI.^13)^ The global number of patients with PAD has increased by 23.5% between 2000 and 2010,^14)^ and there is a concern that the number of PAD patients may increase rapidly in the future. The amputation of the lower limbs not only seriously deteriorates the patient’s quality of life but also causes major social and economic loss.

The currently recommended therapeutic interventions include pain control, risk factor management, and treatment of ulcers or gangrene for CLI and revascularization via bypass surgery or endovascular repair for suitable patients.^15)^ However, revascularization is unsuitable for approximately 25%–40% of CLI patients because they lack the vein grafts needed for the bypass surgery or have multiple extensive artery lesions or comorbidities.^13,16,17)^ The primary goal of the CLI treatment is to save the patient’s life and limbs. Given that prognosis of CLI patients who are not suitable for revascularization or have refractory conditions is very poor, it is socially and medically imperative to develop a treatment strategy for such “no-option” CLI patients.

### Preclinical Studies

Kalka et al.^18)^ obtained EPCs by culturing PB-MNCs from healthy subjects, and transplanted them into the nude mouse model of hindlimb ischemia. When the EPCs were intravenously injected after 2 days of hindlimb ischemia, accumulation of transplanted cells was histologically confirmed at the ischemia site, indicating that they contributed to neovascularization in collaboration with mouse-derived vascular endothelial cells. Cell transplantation significantly improved the capillary density observed histologically and blood perfusion in the ischemic limbs as measured by laser Doppler flowmetry. Consequently, the limb salvage rate from necrosis due to severe ischemia was 59% in the EPC transplantation group, compared with only 7%–8% in the control group. Murohara et al.^19)^ reported similarly positive results of transplantation of human umbilical cord blood EPCs into nude rats with hindlimb ischemia. They obtained the EPCs by culturing human umbilical cord blood MNCs. Losordo et al. also obtained favorable results of administration of granulocyte colony-stimulating factor (G-CSF)-mobilized CD34+ cells obtained from healthy subjects into nude rats with hindlimb ischemia (unpublished data).

### Clinical Studies

**Figure 3:**
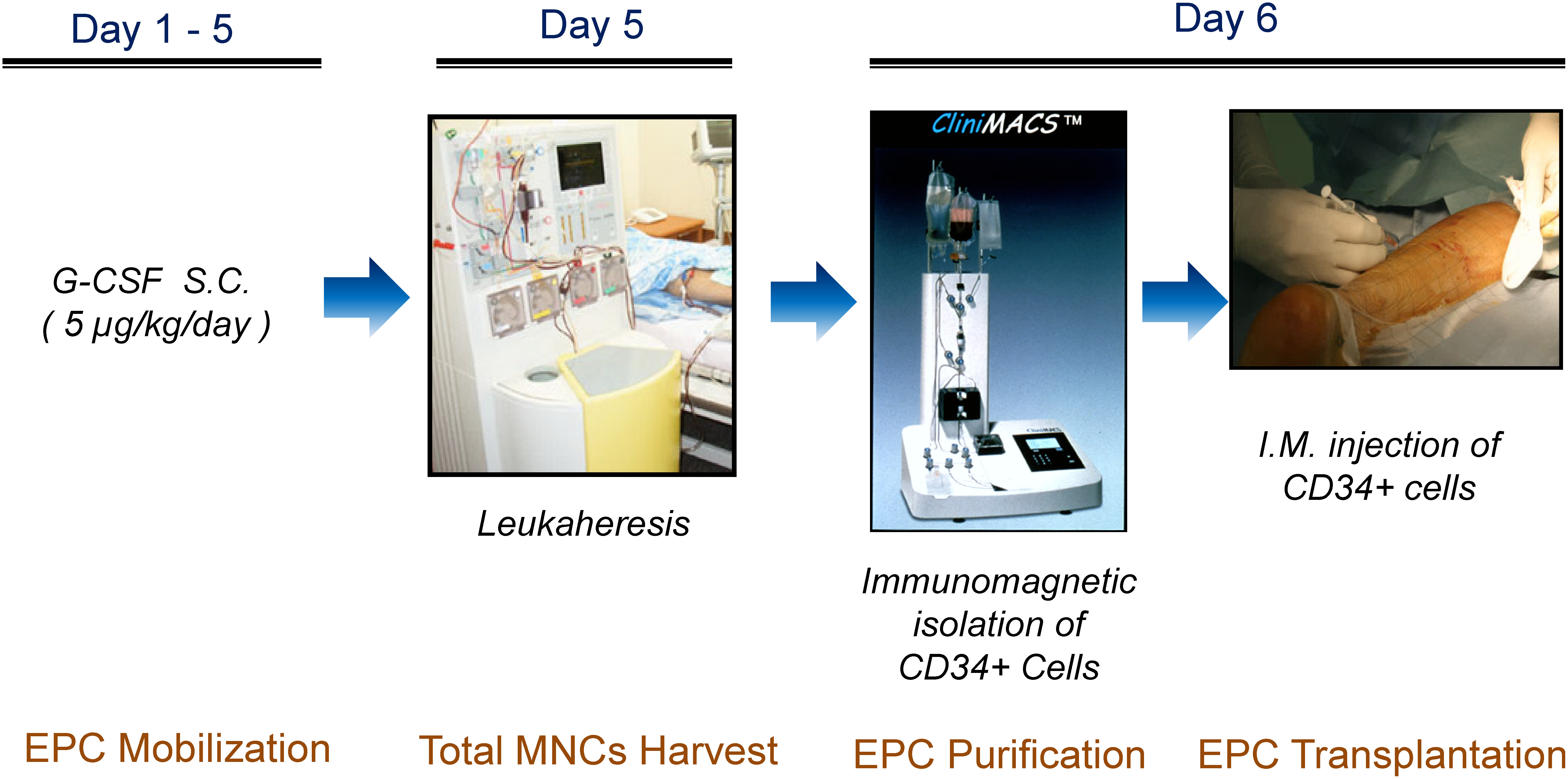
Fig. 3 Mobilization, harvesting, isolation, and transplantation of CD34+ cells in CLI patients.

CLI: critical limb ischemia; EPC: endothelial progenitor cell; G-CSF: granulocyte colony-stimulating factor; I.M.: intramuscular injection; MNC: mononuclear cell; S.C.: subcutaneous injection

Kawamoto et al.^20)^ conducted a multicenter, single-blinded, dose-escalation phase I/IIa clinical trial of intramuscular transplantation of G-CSF-mobilized CD34+ cells in 17 CLI patients (five with ASO, including one on hemodialysis, and 12 with TAO). CD34+ cells were isolated using a magnetic-activated cell sorter, CliniMACS (Miltenyi Biotec, Bergisch Gladbach, Germany) (**Figs. 2** and **3**). No death or major amputation was reported for any patient 1 year following the treatment. The healing of ulcers or necrosis, relief of ischemic pain, and improvement over time with respect to the physiological measures, including toe–brachial pressure index (TBI) and transcutaneous oxygen pressure (TcPO_2_) were observed, and 88% of patients were free of CLI 1 year after the treatment. A long-term follow-up over 4 years following the treatment revealed no major amputation. The proportion of patients free of CLI remained at over 80%. Significant improvement was noted in TBI at 4 years and in TcPO_2_ at 3 years compared with the pretreatment data.^21)^

Fujita et al.^22)^ conducted an investigator-initiated study on 11 CLI patients (seven with TAO and four with ASO) in compliance with good clinical practice for medical devices, aiming a goal of regulatory approval of the CD34+ cell sorter, Isolex (Baxter Healthcare, Deerfield, IL, USA). This study showed favorable safety and efficacy, as had been previously demonstrated in the phase I/IIa study^20,21)^ described above.

Ohtake et al.^23)^ conducted a single-arm, phase II clinical trial of intramuscular transplantation of G-CSF-mobilized CD34+ cells in six CLI patients on dialysis. No serious adverse events related to the treatment were reported in the 1 year following the treatment. The amputation-free survival (AFS) was 100%, and 88% of patients were free of CLI 1 year after the treatment.

Tanaka et al.^24)^ performed a prospective, phase I/IIa clinical trial of G-CSF-mobilized CD34+ cell transplantation for five patients with a nonhealing diabetic foot. All the enrolled patients were on dialysis. No serious adverse events related to the treatment occurred 1 year after the treatment. The wound was completely closed in all the patients at an average of 18 weeks after the treatment.

In Japan, the G-CSF-mobilized CD34+ cell transplantation for CLI patients due to ASO undergoing hemodialysis has been approved as an advanced medical treatment-type B by the Ministry of Health, Labour and Welfare (MHLW) since 2017.

In the United States, Losordo et al.^25)^ initiated a multicenter, randomized, double-blind, placebo-controlled study (ACT34-CLI) of G-CSF-mobilized CD34+ cell transplantation in 28 patients with CLI. The incidence of minor and major amputations tended to be lower in the CD34+ cell group than in the placebo group at 6 and 12 months after the treatment. No adverse events related to cell therapy were reported during the 1 year follow-up period (**Table 1**).

**Table 1:** Table 1 Randomized, double-blind, placebo-controlled clinical study of CD34+ cell therapy for CLI

Trial name or author	Phase	Cell type	Cell dose	Route of administration	Disease severity	Cause of PAD	Number of patients	Follow-up duration	Outcomes	
									Primary endpoints	Other endpoints
ACT34-CLI^25)^	I/IIa	G-CSF-mobilized autologous CD34+ cells	· 1×10^5^ cells/kg · 1×10^6^ cells/kg	IM	Rutherford 4–5	ASO TAO	Total: 28 Cell therapy group: 16 ·1×10^5^ cells/kg: 7 ·1×10^6^ cells/kg: 9 Control group (placebo): 12	1 year	· No cell-related safety issues. · Reduction of major amputation rate in the cell therapy group compared with the placebo group (P=0.125, at 6 months; P=0.058 at 1 year).	

ASO: arteriosclerosis obliterans; CLI: critical limb ischemia; G-CSF: granulocyte-colony stimulating factor; IM: intramuscular; PAD: peripheral arterial disease; TAO: thromboangitis obliterans

In China, Dong et al.^26)^ compared the therapeutic effect of intramuscular administration of G-CSF-mobilized CD34+ cells with G-CSF-mobilized MNCs in 50 patients with CLI caused by TAO (47 patients) and other angiitises (three patients). There were no significant differences in the primary outcome measures of major AFS and total AFS between the CD34+ cell-treated and MNC-treated CLI patients. However, the CD34+ cell-treated patients had a significantly higher probability of rest pain relief than the MNC-treated patients.

Furthermore, based on the positive results from the phase I/IIa clinical study^20,21)^ and the exploratory investigator-initiated study of the medical device,^22)^ a multicenter, randomized, comparative study in patients with CLI, aiming for regulatory approval of CD34+ cells as a regenerative medicine product (ClinicalTrials.gov Identifier: NCT02501018) was initiated in 2017 in Japan. In 2018, this regenerative medicine product was designated by the MHLW as an appropriate product for the Sakigake Strategy, the Scheme for Rapid Authorization of Unapproved Drugs in Japan. The study was completed in May 2022, and the final results are awaited.

## Acute Myocardial Infarction

Primary percutaneous coronary intervention (PCI) after the onset of ST elevation myocardial infarction (STEMI) is widely recognized as the most effective reperfusion modality.^27)^ However, a total of 40%–50% of patients with STEMI develop a microvascular injury, despite successful primary PCI.^28)^ The occurrence of microvascular injury is linked to negative remodeling and left ventricular dysfunction, leading to decreased long-term survival, increased morbidity, and reduced quality of life in comparison with patients with STEMI without microvascular injury.^29)^ Additional strategies following primary PCI are needed to improve the long-term prognosis in this population. For such high-risk patients, cardiovascular regeneration by cell therapy is expected.

### Preclinical Studies

Kocher et al.^30)^ first evaluated the therapeutic effect of intravenous injection of G-CSF-mobilized human CD34+ cells in athymic nude rats with acute myocardial infarction (AMI). They revealed that a single systemic injection of human CD34+ cells after induction of AMI achieved increased capillary density; increased blood flow through CD34+ cell-induced angiogenesis and vasculogenesis in the infarct and peri-infarct zones, which resulted in decreased apoptosis of myocytes in the peri-infarct region; reduced infarct size and collagen deposition; and sustained improvement in cardiac function.

Kawamoto et al.^31)^ intravenously transplanted EPC obtained by culturing healthy human PB-MNCs against an athymic rat myocardial infarction (MI) model. EPC transplantation resulted in increasing capillary density, reducing the left ventricular fibrosis area, and improving left ventricular contractility.

Additionally, Kawamoto et al.^11)^ compared the therapeutic efficacy of G-CSF-mobilized human CD34+ cells with that of unfractionated MNCs in an athymic rat MI model. Despite receiving the same absolute number of CD34+ cells, the MNC group histologically resulted in increased hemorrhagic MI with an abundance of both hematopoietic and inflammatory cells derived from the xenotransplantation, which were not found in the fractionated CD34+ cell group. The CD34+ cell group showed the greatest attenuation of structural changes attributable to the infarct and the greatest improvement of left ventricular function.

### Clinical Studies

As evidence of the superiority of CD34+ cells over MNCs, Hofmann et al.^32)^ conducted a clinical study of intracoronary transplantation of [^18^F]-fluoro-2-deoxy-D-glucose (^18^F-FDG)-labeled unfractionated MNCs or ^18^F-FDG-labeled fractionated CD34+ cells in patients with STEMI who had undergone primary PCI. The subsequent transplanted cell distribution in the body was traced with three-dimensional positron emission tomography. Many of the transplanted MNCs accumulated in the liver and spleen and were also found in the center of the infarcted myocardium, whereas the concentration of the transplanted CD34+ cells was highly specific in the border zone myocardium. These findings suggest that CD34+ cells administered via the coronary artery may efficiently accumulate in the ischemic region and contribute to cardiovascular regeneration.

Based on these nonclinical and clinical studies, Quyyumi et al.^33)^ conducted a phase I dose-escalation study of intracoronary transplantation of autologous BM-derived CD34+ cells in 31 patients with a left ventricular ejection fraction (LVEF) of 50% or less on the fourth day after coronary artery stenting for STEMI. Myocardial perfusion measured by single-photon emission computed tomography (SPECT) improved and myocardial infarct size by gadolinium-enhanced cardiac magnetic resonance imaging (MRI) decreased at 6 months after CD34+ cell transplantation. Dose-dependent effects were also reported (**Table 2**). Following the initial study, a phase II randomized, placebo-controlled study (PreSERVE-AMI study) was performed on 161 patients with an LVEF of 48% or less. Autologous CD34+ cells or placebo was intracoronarily administered on the fourth day after stenting for STEMI.^34)^ There was no significant difference between the two groups in the occurrence of adverse events and in the amount of myocardial perfusion changes by SPECT, which were the primary endpoints, from baseline to 6 months after the treatment. However, a secondary analysis adjusted ischemic time showed significant cell dose-dependent effects on LVEF and infarct size by cardiac MRI (**Table 2**). These results supported the safety of intracoronary transplantation of autologous BM-derived CD34+ cells in patients with left ventricular dysfunction after STEMI and suggested the efficacy.

**Table 2:** Table 2 Randomized-controlled clinical studies of CD34+ cell therapy for STEMI

Trial name or author	Phase	Cell type	Cell dose	Route of administration	Disease severity	Number of patients	Follow-up duration	Outcomes	
								Primary endpoints	Other endpoints
Quyyumi et al.^33)^	I	BM-derived CD34+ cells	· 5×10^6^ cells/body · 10×10^6^ cells/body · 15×10^6^ cells/body	IC	LVEF ≤50%	Total: 31 Cell therapy group: 16 · 5×10^6^ cells: 5 · 10×10^6^ cells: 5 · 15×10^6^ cells: 6 Control group (SOC): 15	6 months	· No significant difference in changes in LVEF measured by MRI between the cell therapy group and the control group from the baseline to 3 or 6 months after treatment. · No cell-related safety issues.	· Reduction of hypoperfusion score measured by SPECT from baseline to 3 or 6 months after treatment in the ≥10×10^6^ cohorts compared with the control group (P=0.02). · Correlation of improved perfusion (P=0.011) and decreased infarct size (P=0.015) with the quantity and mobility of the infused CD34+ cells.
PreSERVE-AMI^34)^	II	BM-derived CD34+ cells	≥10×10^6^ cells/body	IC	LVEF ≤48%	Total: 161 Cell therapy group: 78 Control group (placebo): 83	6 months	· No significant difference in the occurrence of AEs between the cell therapy group and the placebo group. · No significant difference in myocardial perfusion changes by SPECT from baseline to 6 months after treatment between the cell therapy group and the placebo group.	· Cell dose-dependent effect in increase in LVEF (P=0.05) and reduction of infarct size (P=0.02).

AEs: adverse events; BM: bone marrow; IC: intracoronary; LVEF: left ventricular ejection fraction; SOC: standard of care; SPECT: single photon emission computed tomography; STEMI: ST elevation myocardial infarction

## Refractory Angina

Although the mortality rate of coronary artery disease (CAD) has decreased due to advances in coronary revascularization including PCI and bypass surgery and drug therapy, there are 7%–14% of cases that are not indicated for conventional coronary revascularization due to the anatomical form of coronary artery lesions and repeated restenosis and graft occlusion.^35–37)^ Patients with this treatment-resistant angina pectoris have a 1 year mortality rate of 3.9% and a 9 year mortality rate of 28.4%.^38)^ Presently, effective treatment methods for refractory angina have not been established.

### Preclinical Study

Kawamoto et al.^39)^ used a swine chronic myocardial ischemia model to investigate the effect of autologous, nonadhesive PB-CD31+ cells (EPC-rich fraction from total MNCs). Injection of the enriched EPCs into the ischemic myocardium via an injection catheter resulted in an increase in capillary density, a decrease in the ischemic area, and an improvement of LVEF.

### Clinical Studies

The first pilot (phase I/IIa) study of intramyocardial injection of autologous and G-CSF-mobilized CD34+ cells in patients with refractory angina provided early evidence of the feasibility, safety, and bioactivity of the stem cells.^40)^ The promising results encouraged the ACT34-CMI study, a prospective, double-blind, randomized, controlled phase II trial.^41)^ In this study, 167 patients with refractory angina were randomized to receive an intramyocardial injection of 1×10^5^ (low-dose) or 5×10^5^ cells/kg (high-dose) of G-CSF-mobilized autologous CD34+ cells or an equal volume of diluent (placebo). Improvement of weekly angina frequency and exercise tolerance was significantly greater in low-dose patients, but not in high-dose patients, than in placebo-treated patients at 6 and 12 months. Three patients in the control group died in this study. No major adverse cardiovascular events were causatively related to the cell therapy (**Table 3**).

**Table 3:** Table 3 Randomized, double-blind, placebo-controlled clinical studies of CD34+ cell therapy for refractory angina

Trial name or author	Phase	Cell type	Cell dose	Route of administration	Disease severity	Number of patients	Follow-up duration	Outcomes	
								Primary endpoints	Other endpoints
Losordo et al.^40)^	I/IIa	G-CSF mobilized CD34+ cells	· 5×10^4^ cells/kg · 1×10^5^ cells/kg · 5×10^5^ cells/kg	IMC	CCS class III or IV	Total: 24 Cell therapy group: 18 · 5×10^4^ cells/kg: 6 · 1×10^5^ cells/kg: 6 · 5×10^5^ cells/kg: 6 Control group: 6	6 months	· Tendency of reduction in angina frequency, NTG usage, and CCS from baseline to 6 months after treatment in the cell therapy group compared with the control group. · No cell-related safety issues.	
ACT34-CMI^41)^	II	G-CSF mobilized CD34+ cells	· 1×10^5^ cells/kg · 5×10^5^ cells/kg	IMC	CCS class III or IV	Total: 167 Cell therapy group: 111 · 1×10^5^ cells/kg (low-dose): 55 · 5×10^5^ cells/kg (high-dose): 56 Control group: 56	12 months	· Weekly angina frequency was significantly lower in the low-dose group than in the control group (P=0.02, at 6 months; P=0.035, at 24 months after treatment).	· Improvement in ETT in the low-dose group compared with the control group (P=0.014, at 6 months; P=0.017, at 24 months after treatment).
RENEW^44)^	III	G-CSF mobilized CD34+ cells	1×10^5^ cells/kg	IMC	CCS class III or IV	Total: 112 Cell therapy group: 57 Placebo group: 27 SOC group: 28	12 months	· No significant difference in changes in ETT from baseline to 12 months after treatment between the cell therapy group and the placebo group.	· Angina frequency at 6 months after treatment was significantly in the cell therapy group than in the placebo group (P=0.02).

CCS: Canadian Cardiovascular Society; ETT: exercise tolerance test; G-CSF: granulocyte colony-stimulating factor; IMC: intramyocardial; NTG: nitroglycerine; SOC: standard of care

Subsequently, a 24 month follow-up study of ACT34-CMI patients revealed that weekly angina frequency at 24 months was significantly reduced in patients treated with both low- and high-dose CD34+ cells compared with placebo-treated patients. The incidence of major adverse cardiac events (MACE; MI, acute coronary syndrome, or heart failure and hospitalization) and mortality tended to be lower in both cell-treated groups than in the placebo group.^42)^

Furthermore, in the RENEW (Efficacy and Safety of Targeted Intramyocardial Delivery of Auto CD34+ Stem Cells for Improving Exercise Capacity in Subjects with Refractory Angina) trial, a randomized, double-blind, multicenter phase III trial,^43,44)^ although the trial was terminated for the sponsor’s strategic considerations after enrollment of 112 of the planned 444 patients, a total of 112 patients with no-option refractory angina were allocated to either open-label standard of care (SOC) group or double-blinded active control (AC) group in which patients underwent subcutaneous injection of G-CSF, leukapheresis and intramyocardial injection of placebo, or 1×10^5^ cells/kg of CD34+ cells. There was no statistically significant difference in the primary endpoint, change in total exercise time (TET) at 12 months after treatment between the cell therapy group and AC group; however, angina frequency at 6 months, which was one of the secondary endpoints, was improved in the cell therapy group compared with the AC group. The incidence of MACE including all-cause death, MI, stroke, or cardiovascular hospitalization is the highest in the SOC group and comparable between AC and cell therapy groups (**Table 3**).

A meta-analysis of these three randomized double-blinded trials revealed that autologous CD34+ cell therapy significantly improved change in TET at 3 and 6 months and tended to improve change in TET at 12 months, and the relative frequency of angina was significantly lower at 3, 6, and 12 months in the CD34+ cell therapy group than in the placebo group. As for safety, autologous CD34+ cell therapy significantly decreased both mortality and incidence of MACE including all-cause death, MI, stroke, or cardiovascular hospitalization at 24 months.^45)^

## Ischemic Cardiomyopathy

Ischemic cardiomyopathy (ICM) refers to a condition in which the left ventricular myocardium is injured due to CAD, followed by pathological fibrosis at the injury site, resulting in a decrease in LVEF.^46–48)^ Based on the results of nonclinical studies introduced in the AMI and Refractory Angina sections, CD34+ cell therapy for ICM has been attempted.

### Clinical Study

Poglajen et al.^49)^ conducted the first prospective crossover study to investigate the clinical effects of intramyocardial injection of G-CSF-mobilized CD34+ cells in patients with ICM with New York Heart Association (NYHA) class III and LVEF of <40%. In the phase I study, 33 patients were treated with medical therapy for 6 months, subsequently in the phase II study, 31 patients (two patients died during the phase I study) underwent intramyocardial injection of CD34+ cells and were followed up for 6 months after the treatment. There were no significant changes in LVEF, N-terminal pro-B-type natriuretic peptide (NT-proBNP), or 6 min walk distance in phase I, whereas in phase II, LVEF, NT-proBNP, and 6 min walk distance were significantly improved from baseline to 6 months.

## Nonischemic Dilated Cardiomyopathy

Nonischemic dilated cardiomyopathy (DCM) is characterized via myocardial dilatation and dysfunction in the absence of CAD, hypertension, valvular heart disease, or congenital heart disease. The etiology of nonischemic DCM is multifactorial; moreover, genetic, infectious, and toxicity-related factors and mechanical stress can lead to the development of nonischemic DCM.^50)^ Additionally, current preclinical and clinical studies have presented evidence that marked vascular derangements and impaired vascularization and angiogenesis can be related to the progression of nonischemic DCM.^51)^ Hence, therapeutic strategies aimed at repairing vasculogenesis and angiogenesis also may benefit patients with nonischemic DCM.

### Clinical Studies

Vrtovec et al.^52)^ conducted the phase I clinical study assessing the effects of G-CSF-mobilized CD34+ cells on DCM. Fifty-five patients with DCM with LVEF <30% and NYHA class III were randomly assigned to 28 patients who received an intracoronary injection of CD34+ cells (the cell treatment group) or 27 patients who did not receive cell therapy (the control group). At 1 year, LVEF as determined via echocardiography and 6 min walk distance were significantly increased, and N-terminal probrain natriuretic peptide (NT-pro-BNP) was significantly decreased in the cell treatment group compared with the control group. The combined mortality or heart transplantation rate was lower in the cell treatment group than in the control group during 1 year follow-up period (**Table 4**). Additionally, these positive effects persisted up to 5 years after cell therapy.^53)^ Furthermore, this group presented that intracoronary injection of G-CSF-mobilized CD34+ cells might improve myocardial perfusion as defined by scintigraphy as well as LVEF and 6 min walk at 6 months after CD34+ cell therapy.^54)^

**Table 4:** Table 4 Randomized-controlled clinical studies of CD34+ cell therapy for DCM

Trial name or author	Phase	Cell type	Cell dose	Route of administration	Disease severity	Number of patients	Follow-up duration	Outcomes	
								Primary endpoints	Other endpoints
Vrtovec et al.^52)^	I	G-CSF mobilized CD34+ cells	(123±23)×10^6^ cells/body	IC	LVEF<30% NYHA≥III	Total: 55 Cell therapy group: 28 Control (SOC) group: 27	12 months	· Increase in LVEF from baseline to 12 months after treatment in the cell therapy group compared with the control group (P=0.03). · Increase in 6 min walk distance from the baseline to 12 months after treatment in the cell therapy group compared with the control group (P=0.001).	· Decrease in NT-proBNP from baseline to 12 months after treatment in the cell therapy group compared with the control group (P=0.01). · One-year mortality or heart transplantation was lower in the cell therapy group compared with the control group at 12 months after treatment (P=0.03).
Vrtovec et al.^55)^	II	G-CSF mobilized CD34+ cells	· (105±31)×10^6^ cells/body (IMC group) · (103±27)×10^6^ cells/body (IC group)	· IMC · IC	LVEF<40% NYHA≥III	Total: 40 IMC group: 20 IC group: 20	6 months	· Increase in LVEF from baseline to 6 months after treatment in the IMC group compared with the IC group (P=0.03).	· Increase in 6 min walk distance from the baseline to 6 months after treatment in the IMC group compared with the IC group (P=0.03). · Decrease in NT-proBNP from baseline to 6 months after treatment in the IMC group compared with the IC group (P=0.04).
REMEDIUM^56)^	II/III	G-CSF mobilized CD34+ cells	80×10^6^ cells/body	IMC	LVEF<40% NYHA III	Total: 60 Repetitive cell therapy group (Group A): 30 Single-cell therapy group (Group B): 30	12 months	· No significant difference in change in LVEF from baseline to 12 months after treatment between group A and group B.	· No significant difference in changes in LVEDD, NT-proBNP, and 6 min walk distance from the baseline to 12 months after treatment between groups A and B.

DCM: dilated cardiomyopathy; G-CSF: granulocyte colony-stimulating factor; IC: intracoronary; IMC: intramyocardial; LVEDD: left ventricular end-diastolic dimension; LVEF: left ventricular ejection fraction; NT-proBNP: N-terminal probrain natriuretic peptide; NYHA: New York Heart Association; SOC: standard of care

To investigate whether intramyocardial injection of G-CSF-mobilized CD34+ cells is associated with greater cell retention rates and clinical improvement than an intracoronary injection of CD34+ cells, an open-label study was conducted in 40 patients with nonischemic DCM. Twenty patients were randomized to receive an intracoronary injection (the IC group) and 20 received an intramyocardial injection of CD34+ cells (the IMC group).^55)^ In both groups, CD34+ cells were labeled with a technetium-99 m radioisotope for SPECT imaging. In the IC group, cells were injected by the intracoronary route into the artery supplying segments of greater perfusion defect as shown on myocardial perfusion scintigraphy. In the IMC group, electromechanical mapping was used to identify viable but dysfunctional myocardium, and intramyocardial injection was performed. At 18 h after the procedure, myocardial retention quantified by SPECT was higher in the IMC group than in the IC group (P<0.01). At 6 months after treatment, LVEF, 6 min walk distance and NT-pro-BNP level significantly improved in the IMC group compared with the IC group. These results revealed that IMC injection of CD34+ cells was associated with higher myocardial retention rates and greater improvement in left ventricular function (**Table 4**).

Recently, this research group performed the REMEDIUM study, a phase II/III trial to investigate the effects of repetitive intramyocardial injection of CD34+ cells in patients with nonischemic DCM.^56)^ Sixty patients (LVEF <40%, NYHA class III) were randomly allocated in a 1 : 1 ratio to receive either repetitive or single-dose CD34+ cell therapy. However, this study could not present the superiority of repetitive CD34+ cell administration in improvements of LVEF, NT-proBNP, or 6 min walk distance at 1 year compared with single-dose cell therapy (**Table 4**).

## Cerebrovascular Disease

### Preclinical Studies

Taguchi et al.^57)^ first investigated the therapeutic effect of intravenous (iv) injection of CD34+ cells isolated from human umbilical cord blood to severe combined immunodeficient (SCID) mice subjected to permanent cerebral ischemia induced by ligation of the left middle cerebral artery (MCA) 48 h prior to the administration of cells. The administration of CD34+ cells after stroke significantly induced neovascularization in the peri-infarct zone, enhanced blood flow just outside of the penumbra, and induced cortical expansion, accompanied by neurological functional recovery assessed by behavioral analysis compared with administration of CD34− cells and phosphate buffer saline (PBS).

Ogawa et al.^58)^ compared the therapeutic effect among iv injection of G-CSF-mobilized human CD34+ cells, intracarotid arterial (ia) injection of G-CSF-mobilized human CD34+ cells, and iv injection of PBS in SCID mice 4 weeks after permanent ligation of the MCA. At 8 weeks after injection, indicators of behavioral tests including wire hang test and rotarod test were significantly better in the ia injection group than in the iv injection group and the PBS group.

These results suggest that the administration of CD34+ cells may have a therapeutic effect on both acute and chronic phases of ischemic stroke.

### Clinical Studies

Banerjee et al.^59)^ conducted a first pilot study of autologous CD34+ cell therapy in five patients with acute ischemic stroke (within 7 days of onset) and the National Institute of Health Stroke Scale (NIHSS) score ≥8. CD34+ cells were collected from the BM of the patient, positively selected using an immune-magnetic cell selection device, and delivered via the MCA on the affected side. All patients showed improvements in the modified Rankin scale (mRS) score and NIHSS score during the 6 month follow-up period.

Chen et al.^60)^ performed a randomized, single-blind controlled study of stereotaxic implantation of G-CSF-mobilized CD34+ cells in patients with chronic cerebral infarction (6 months to 5 years after a stroke). Thirty patients were randomized into the cell treatment group or the control group. All 30 patients completed the 12 month follow-up. No serious adverse events occurred during the study period. Improvements in stroke scales including the NIHSS, the European stroke scale (ESS) and ESS motor scale, and the mRS (functional outcome scale) from baseline to the end of the 12 month follow-up period were significantly greater in the cell treatment group than in the control group (**Table 5**).

**Table 5:** Table 5 Randomized-controlled clinical study of CD34+ cell therapy for chronic ischemic stroke

Trial name or author	Phase	Cell type	Cell dose	Route of administration	Number of years after the onset of ischemic stroke	Disease severity	Number of patients	Follow-up duration	Outcomes	
									Primary endpoints	Other endpoints
Chen et al.^60)^	II	G-CSF mobilized CD34+ cells	3–8×10^6^ cells/body	stereotaxic implantation	0.5–5	NIHSS: 9–20	Total: 30 Cell therapy group: 15 Control (SOC) group: 15	12 months	· Improvement in NIHSS in the cell therapy group compared with the control group from baseline to 12 months after treatment (P=0.0002).	· Improvement in ESS (P=0.005) and EMS (P=0.009) in the cell therapy group compared with the control group from baseline to 12 months after treatment. · Improvement in mRS in the cell therapy group compared with the control group from baseline to 12 months after treatment (P=0.004). · No serious adverse events during the study period.

EMS: European stroke motor subscale; ESS: European stroke scale; G-CSF: granulocyte colony-stimulating factor; mRS: modified Rankin scale; NIHSS: National Institutes of Health Stroke Scale; SOC: standard of care

In Japan, a randomized, double-blind, placebo-controlled clinical study of intracarotid injection of autologous G-CSF-mobilized CD34+ cells in chronic ischemic stroke (Japan Registry of Clinical Trials Identifier: jRCT2052200112) started in 2020, and the subject recruitment is still ongoing.

## Conclusions and Future Perspectives

Since the discovery in 1997 that CD34+ cells in human PB-MNCs are the EPC-enriched fraction, preclinical and clinical studies have been steadily progressed worldwide to accumulate pieces of evidence suggesting the safety and efficacy of CD34+ cell therapy. Particularly, a multicenter randomized controlled trial in CLI patients was started in December 2017, 20 years after the discovery of EPC, with the aim of regulatory approval of CD34+ cells as a regenerative medicine product in Japan. This regenerative medicine product has been designated as an appropriate product for the Sakigake Strategy. If this product is pharmaceutically approved in the future, it is expected to be a stepping stone for expanding indications for diseases other than CLI in Japan. Additionally, in the United States, CD34+ cell product has received Regenerative Medicine Advanced Therapy designation by Food and Drug Administration for refractory angina. Hence, overseas development of CD34+ cell therapy is also attracting attention.

Recently, CD34+ cell therapy has also been applied to fracture nonunion^61)^ and decompensated liver cirrhosis,^62)^ in which reduced blood supply is a key factor associated with disease progression. Both preclinical and pilot clinical studies demonstrated the safety, feasibility, and effectiveness of G-CSF-mobilized CD34+ cell therapy in these diseases. The application of CD34+ cells to such nonvascular diseases is also an attractive and novel therapeutic option for unmet medical needs.
